# Perceived role isolation and work addiction risk among entrepreneurs: associations with perceived business success

**DOI:** 10.3389/fpsyg.2026.1968268

**Published:** 2026-09-23

**Authors:** Attila L. Makai, Tibor Dőry

**Affiliations:** Department of International and Applied Economics, Széchenyi István University, Gyor, Hungary

**Keywords:** entrepreneurial role isolation, entrepreneurial wellbeing, perceived business success, work addiction, work passion

## Abstract

**Introduction:**

Entrepreneurs and business leaders evaluate the condition of their businesses partly through self-referential judgments rather than financial indicators alone. Little is known, however, about how such judgments relate to maladaptive work investment and to the social experience of the leadership role. This study examines associations among work addiction risk, perceived entrepreneurial role isolation, recovery-related leisure time, work passion, and perceived business success.

**Methods:**

The evidence comes from a cross-sectional sample of 184 Hungarian entrepreneurs and business leaders, all of whom held an ownership stake in the business they led. Perceived business success was operationalized as a composite of self-rated business performance, revenue growth, and competitor-relative performance. Associations were examined with Spearman correlations, group comparisons, and hierarchical regression models, complemented by robustness checks and bootstrapped indirect-association analyses.

**Results:**

Work addiction risk was negatively associated with perceived business success at the bivariate level and differentiated an adverse functioning profile involving higher stress, burnout exhaustion, and obsessive passion. In multivariate models, however, perceived role isolation was the most consistent correlate of lower perceived business success, whereas work addiction risk was not. The isolation association remained significant across alternative outcome specifications, distress controls, firm-level covariates, and data-screening exclusions. Exploratory analyses supported an indirect association between work addiction risk and perceived business success through role isolation, but not through recovery-related leisure time.

**Discussion:**

The findings position perceived role isolation as a central psychosocial correlate of business self-evaluation and connect perceived entrepreneurial success research with occupational health psychology. Given the cross-sectional design, all associations are interpreted as non-causal.

## 1 Introduction

Whether working more makes entrepreneurs feel more successful is less self-evident than everyday intuition suggests. Entrepreneurs and business leaders often treat long hours, constant availability, and total immersion as signals of commitment, and such signals are socially rewarded in many business cultures. Research on work addiction nonetheless indicates that heavy work investment can take an excessive and compulsive form. Involvement of this kind is difficult to regulate and tends to be accompanied by strain rather than accomplishment (Andreassen et al., 2012; Schaufeli et al., 2009; Clark et al., 2016). Entrepreneurship research has established, at the same time, that success is partly a self-referential judgment. Entrepreneurs evaluate achievement against personally meaningful criteria that include, but are not limited to, financial performance (Wach et al., 2016, 2020; Walker and Brown, 2004; Gorgievski et al., 2011). How these two literatures intersect remains empirically unresolved. It is not known whether maladaptive work investment is associated with less favourable business self-evaluations, nor through which role conditions any such association runs.

This question matters because entrepreneurial work is not a scaled-down version of employment. Research on entrepreneurs’ mental health has argued that entrepreneurial activity is embedded in distinctive decision-making, identity, uncertainty, and responsibility structures. Employee-based models therefore cannot simply be transferred to business owners and founders (Stephan, 2018; Wiklund et al., 2019). Three role conditions are particularly salient. The entrepreneurial role concentrates responsibility: strategic decisions, financial exposure, and employment consequences may rest on a single person who has no peers or superiors with whom that responsibility is shared. The social experience of the role, including perceived isolation, is therefore analytically consequential rather than incidental (Fernet et al., 2016; Umdasch and Berger, 2025). Business-related cognition also extends beyond formal working time. Entrepreneurs may continue processing customers, cash flow, and operational problems during evenings and weekends, which places recovery and psychological detachment at the centre of entrepreneurial functioning (Sonnentag and Fritz, 2015; Wach et al., 2021). Strong identification with the venture blurs, in addition, the boundary between adaptive commitment and compulsive overinvestment, a distinction that the dualistic model of passion renders theoretically tractable (Vallerand et al., 2003; Newman et al., 2020).

The present study examines perceived business success in relation to work addiction risk, perceived entrepreneurial role isolation, recovery-related leisure time, and harmonious and obsessive passion. The evidence comes from a cross-sectional sample of 184 Hungarian entrepreneurs and business leaders, all of whom held an ownership stake in the business they led. Perceived business success is deliberately defined more narrowly than full multidimensional subjective entrepreneurial success. It comprises self-rated business performance, revenue development, and performance relative to competitors, and thus corresponds most closely to the firm-performance facet of subjective success frameworks (Wach et al., 2016, 2020). This restriction is a design decision rather than a limitation of ambition. It keeps the outcome anchored in economic and business-related self-evaluation, where the tension between working more and evaluating the business more favourably is most direct.

The findings qualify a simple account in which work addiction translates directly into lower perceived success. Work addiction risk was negatively associated with perceived business success at the bivariate level and distinguished an adverse functioning profile, but the association did not survive full adjustment. Perceived role isolation showed the opposite pattern of stability. It was negatively associated with perceived business success across bivariate, multivariate, and robustness analyses, including models that controlled for firm size, ownership share, life-cycle stage, leadership position, and industry. An exploratory indirect association between work addiction risk and perceived business success through role isolation was supported; an analogous pathway through recovery-related leisure time was not.

The study contributes to the literature in three ways. It extends perceived entrepreneurial success research by testing whether maladaptive work investment relates to business self-evaluation, and it shows that this relation is better understood as part of a broader role-functioning profile than as a stable direct association. It positions perceived entrepreneurial role isolation as a robust correlate of perceived business success, one that was not accounted for by the distress-related, motivational, and firm-level covariates available in this study. The social experience of the leadership role thereby gains a more central place in success research than it has typically occupied. The study also connects entrepreneurship research with occupational health psychology by locating work addiction risk, recovery, and passion within a single empirical model of business self-evaluation. It does so while maintaining the conceptual distinction between perceived economic and business success and broader subjective entrepreneurial success.

## 2 Literature review and hypothesis development

### 2.1 Perceived business success and subjective entrepreneurial success

Entrepreneurial success has long been assessed through externally observable indicators such as profit, turnover, employment growth, sales growth, and survival. These indicators remain important, but they provide only a partial account of how entrepreneurs and business leaders understand success. Success judgments are embedded in personally meaningful goals, values, and role expectations, and entrepreneurs do not necessarily evaluate achievement only in financial terms. This argument is central to the literature on subjective entrepreneurial success, which defines success as the entrepreneur’s own assessment of achievement against criteria that are important to them (Wach et al., 2016, 2020).

Wach et al. (2016) conceptualized subjective entrepreneurial success as a multi-factorial construct comprising firm performance, workplace relationships, personal fulfilment, community impact, and personal financial rewards. This framework matters for the present study because it differentiates economic from non-economic success dimensions without dismissing business performance. The firm-performance facet remains a key component of subjective success, but it is no longer the only meaningful criterion. Wach et al. (2020) extended this line of work to entrepreneurs’ achieved success and again emphasized that self-evaluations span multiple domains. Small business research supports the broader view. Walker and Brown (2004) showed that owners use both financial and lifestyle-related criteria when assessing success. Gorgievski et al. (2011) linked owners’ success criteria to value orientations, with self-enhancement values emphasizing growth and profitability and self-transcendent values emphasizing stakeholder satisfaction and work-life balance.

The present study does not claim to measure full multidimensional subjective entrepreneurial success. The available indicators are self-rated business performance, revenue development, and competitor-relative performance. Together they capture a narrower construct, perceived economic or business success, corresponding most closely to the firm-performance facet of subjective success frameworks. Prior evidence that subjective success facets relate systematically to quasi-objective and objective indicators supports the use of perceived performance measures while underscoring the need for conceptual precision (Wach et al., 2016, 2020). Dijkhuizen et al. (2018) demonstrated that entrepreneurs’ wellbeing predicts later subjective success but not objective business performance, which indicates that subjective and objective success indicators should not be treated as interchangeable. Perceived business success is therefore positioned here as a legitimate but bounded outcome. It does not substitute for objective performance data, nor does it exhaust the domain of subjective entrepreneurial success, but it captures how entrepreneurs and business leaders evaluate the economic and competitive condition of their business. This precision allows the study to ask whether such evaluations are associated not only with conventional business or motivational factors but also with maladaptive work investment, perceived role isolation, and recovery-related functioning.

### 2.2 Work addiction risk as maladaptive work investment

Entrepreneurs and business leaders often engage in substantial work investment. High involvement may support persistence, innovation, and firm development, but the literature on workaholism and work addiction cautions against equating all forms of heavy work investment with adaptive engagement. Work addiction refers to excessive and compulsive work involvement that is difficult to control. Adverse psychological, interpersonal, and health-related outcomes are among its documented correlates rather than part of its definition (Andreassen et al., 2012; Clark et al., 2016). Two measurement traditions are especially relevant. The Bergen Work Addiction Scale is grounded in addiction components, namely salience, tolerance, withdrawal, conflict, relapse, mood modification, and problems, and provides a concise instrument for assessing work addiction risk (Andreassen et al., 2012). The Dutch Workaholism Scale conceptualizes workaholism as working excessively and working compulsively, distinguishing behavioural intensity from cognitive compulsion (Schaufeli et al., 2009). Both approaches converge on the view that maladaptive work investment is more than long working hours; it is rigid, compulsive, and potentially impairing involvement in work.

Meta-analytic evidence links workaholism to job stress, burnout, work-life conflict, and poorer physical and mental health (Clark et al., 2016). Such evidence challenges the assumption that working more necessarily produces better outcomes. Entrepreneurship-specific findings add nuance. Gorgievski et al. (2014) found that work engagement and workaholism relate differently to entrepreneurial performance through affective pathways. Engagement was linked to positive affect and favourable performance indicators, whereas workaholism was linked to negative affect, which in turn related negatively to business growth and subjective business success; both nonetheless showed positive direct associations with innovative behaviour. More recent evidence from business owners and family businesses complicates the assumption that additional work pays off. Wiatt et al. (2024) found that compulsive work among small business owners did not translate into higher business income. Clark et al. (2025) linked workaholism in family business leaders to after-hours technology use and to work interference with family. Compulsive work investment may thus be accompanied by strain and boundary intrusion without producing superior economic outcomes.

On this basis, work addiction risk is expected to be negatively associated with perceived business success. The argument is not that work addiction necessarily reduces objective performance. It is that compulsive work investment may be accompanied by strain, negative affect, and self-evaluative patterns that relate to lower perceived business success.

*H1*. Higher work addiction risk will be associated with lower perceived business success.

### 2.3 Perceived entrepreneurial role isolation

Entrepreneurship is frequently described as autonomous, agentic, and self-directed. Autonomy in business leadership, however, may also entail role-based isolation. Entrepreneurs and business leaders carry responsibility for strategic decisions, financial risks, employment consequences, and business continuity. In owner-managed or small business contexts these responsibilities are often not shared with peers or superiors. The social experience of entrepreneurship therefore deserves attention alongside motivation and performance.

The emerging literature on entrepreneurs’ loneliness provides the conceptual foundation. Loneliness is generally understood as a subjective emotional state arising from perceived insufficiency in the quality or quantity of social connections. Umdasch and Berger (2025) emphasize the need for construct clarity in entrepreneurship, leadership, and workplace loneliness research, because loneliness, isolation, lack of support, and social embeddedness are related but not identical constructs. This distinction informs the terminology adopted here. The study refers to perceived entrepreneurial role isolation rather than loneliness, because the available measure captures whether respondents felt isolated or surrounded in their managerial role and should be understood as a role-specific proxy rather than a validated loneliness scale.

At least three mechanisms make role isolation a plausible correlate of business self-evaluation, and distinguishing them clarifies what the present design can and cannot adjudicate. An informational mechanism operates through feedback. Leaders who lack trusted interlocutors, such as peers, mentors, or advisory relationships, receive less calibrating feedback about how their business is performing, and their self-evaluations may remain anchored in worry rather than comparison. The relevance of relational resources to success judgments is consistent with evidence that perceived entrepreneurial success is embedded in social-relational conditions such as interpersonal influence and workplace relationships (Aguinis et al., 2008; Wach et al., 2016, 2020). An affective mechanism runs through the aversiveness of isolation itself. The resulting negative affect may colour evaluations of the business, a pathway consistent with evidence that occupational loneliness participates in stressor-burnout processes among SME owner-managers (Fernet et al., 2016). An instrumental mechanism concerns practical support. Isolated leaders may lack the assistance that would otherwise buffer demands, so that the condition of the business, and not only its evaluation, may be affected. This possibility is suggested by evidence linking entrepreneurs’ loneliness to business exit intentions through reduced entrepreneurial passion (Zhu et al., 2023). The cross-sectional design of the present study cannot separate these mechanisms or establish direction. What it can establish is whether the association between role isolation and perceived business success is robust to distress, motivational, and firm-level alternatives, which is a precondition for any of the mechanisms to be worth pursuing.

Accordingly, perceived entrepreneurial role isolation is expected to be negatively associated with perceived business success. Work addiction risk may in addition be positively related to role isolation. Excessive and compulsive work may reduce social availability, intensify role immersion, and narrow attention to business-related demands; conversely, isolated leaders may invest more compulsively in work as a compensatory or coping response. Given the cross-sectional design, the study tests association rather than causal direction.

*H2*. Higher perceived entrepreneurial role isolation will be associated with lower perceived business success.

*H3*. Higher work addiction risk will be associated with higher perceived entrepreneurial role isolation.

### 2.4 Recovery-related leisure time and work-related cognition

Recovery from work is a core concept in occupational health psychology. Psychological detachment refers to mentally disengaging from work during nonwork time and refraining from work-related thoughts and activities. The stressor-detachment model proposes that work stressors, especially workload, reduce detachment, and that low detachment is associated with higher strain and poorer wellbeing, with detachment functioning as mediator or moderator in stressor-strain processes (Sonnentag and Fritz, 2015). Recovery reviews similarly show that detachment and other recovery experiences are associated with lower exhaustion and higher wellbeing, while high demands tend to undermine recovery (Sonnentag et al., 2018).

Recovery is particularly relevant for entrepreneurship because business-related cognition extends beyond formal working time. Wach et al. (2021) provided diary evidence that entrepreneurial stressors can impair evening detachment through problem-solving pondering and affective rumination, with consequences for next-morning wellbeing. Their study situates detachment within entrepreneurial work rather than relying on employee samples alone. Work-related thinking is not uniformly detrimental, however. Querstret and Cropley (2012) distinguished affective rumination, problem-solving pondering, and detachment, and showed that these forms of work-related cognition have different implications for fatigue and sleep. Affective rumination appears more detrimental, whereas problem-solving pondering may be less harmful or even functional. This distinction is essential for interpreting the present measure, which does not capture validated psychological detachment or rumination quality. The measure instead records recovery-related leisure time, that is, non-sleep leisure time during which respondents report not thinking about business-related tasks, and it is used as a proxy indicator of detachment-related recovery capacity.

Because work addiction risk involves persistent work-related salience and compulsion, it is expected to be associated with lower recovery-related leisure time. Entrepreneurs and business leaders with higher work addiction risk may find it more difficult to disengage cognitively from work even during nominal leisure. The relationship between recovery-related leisure time and perceived business success is less certain. Recovery is robustly linked to wellbeing and strain, but its direct association with perceived economic and business success is less established. Recovery-related leisure time is therefore treated as central to the work addiction profile, while its role in perceived business success is examined more cautiously.

*H4*. Higher work addiction risk will be associated with lower recovery-related leisure time.

### 2.5 Harmonious and obsessive passion

Work passion offers a motivational lens for distinguishing adaptive commitment from compulsive overinvestment. The dualistic model of passion defines passion as a strong inclination toward an activity that individuals like, find important, and invest time and energy in. The model distinguishes harmonious passion, grounded in autonomous internalization, from obsessive passion, associated with controlled internalization and rigid persistence (Vallerand et al., 2003). Harmonious passion allows the activity to remain integrated with other life domains. Obsessive passion is more likely to be accompanied by rigidity, conflict, and difficulty disengaging. Obsessive passion and work addiction are conceptually adjacent, since both involve rigid and difficult-to-regulate involvement. The former is nevertheless defined by the controlled internalization of a valued activity into identity, whereas the latter is defined by addiction-like symptomatology. They are therefore treated here as related but distinct constructs, and their empirical association is reported rather than assumed.

The distinction is particularly relevant in entrepreneurship, where strong identification with the venture can be productive but may also blur the boundary between commitment and compulsion. Reviews of entrepreneurial passion indicate that the field requires more careful attention to measurement, outcomes, and the distinction between adaptive and maladaptive motivational forms (Newman et al., 2020). The broader passion literature provides grounds for differentiated expectations. Vallerand et al. (2003) linked harmonious passion to more adaptive functioning and obsessive passion to more rigid involvement. Vallerand (2008) connected obsessive passion to negative affect and rumination when engagement in the passionate activity is blocked. Meta-analytic evidence suggests that harmonious passion is more consistently associated with positive outcomes, whereas obsessive passion shows mixed associations, including links with rumination and negative affect (Curran et al., 2015).

On this basis, three component expectations follow, and they are stated as separate hypotheses to keep each claim testable on its own terms. Harmonious passion is expected to be positively associated with perceived business success, because autonomous and integrated work involvement should align with more favourable self-evaluation (Vallerand et al., 2003; Curran et al., 2015). Obsessive passion is expected to be positively associated with work addiction risk, because controlled and rigid involvement overlaps with compulsive work investment in its difficulty of disengagement, even though the two constructs remain conceptually distinct (Vallerand et al., 2003). Obsessive passion is further expected to be negatively associated with recovery-related leisure time, because obsessive engagement has been linked to rumination and impaired disengagement when the activity is set aside (Vallerand, 2008; Curran et al., 2015).

*H5a*. Harmonious passion will be positively associated with perceived business success.

*H5b*. Obsessive passion will be positively associated with work addiction risk.

*H5c*. Obsessive passion will be negatively associated with recovery-related leisure time.

### 2.6 Integrative model and exploratory propositions

The reviewed literature suggests that perceived business success is shaped by more than financial conditions or work intensity alone. Entrepreneurs’ self-evaluations are embedded in motivational, relational, and recovery-related conditions. Work addiction risk captures maladaptive work investment, and perceived role isolation captures the social experience of the entrepreneurial role. Recovery-related leisure time captures a proxy for cognitive disengagement from business concerns, and passion differentiates adaptive from rigid motivational involvement.

The central integrative question is whether work addiction risk is directly associated with perceived business success, or whether this association is better understood as part of a broader profile involving perceived role isolation and impaired recovery. The role-isolation pathway is theoretically plausible because work addiction risk may be associated with a more isolated role experience, and isolation is expected to relate to lower perceived business success. The recovery pathway is also plausible but more tentative, because detachment is strongly linked to wellbeing and strain, whereas its direct connection to perceived business success remains underdeveloped. Given the cross-sectional data, these relationships are examined as exploratory indirect associations rather than causal mediation. Temporal ordering cannot be established, and cross-sectional estimates of longitudinal mediation processes can be biased (Maxwell and Cole, 2007; O’Laughlin et al., 2018). The two exploratory propositions are therefore stated in the form in which they are tested: as expected attenuation of the focal association when a candidate intervening variable is added, together with a bootstrapped estimate of the corresponding indirect association (Figure 1).

**Figure 1:**
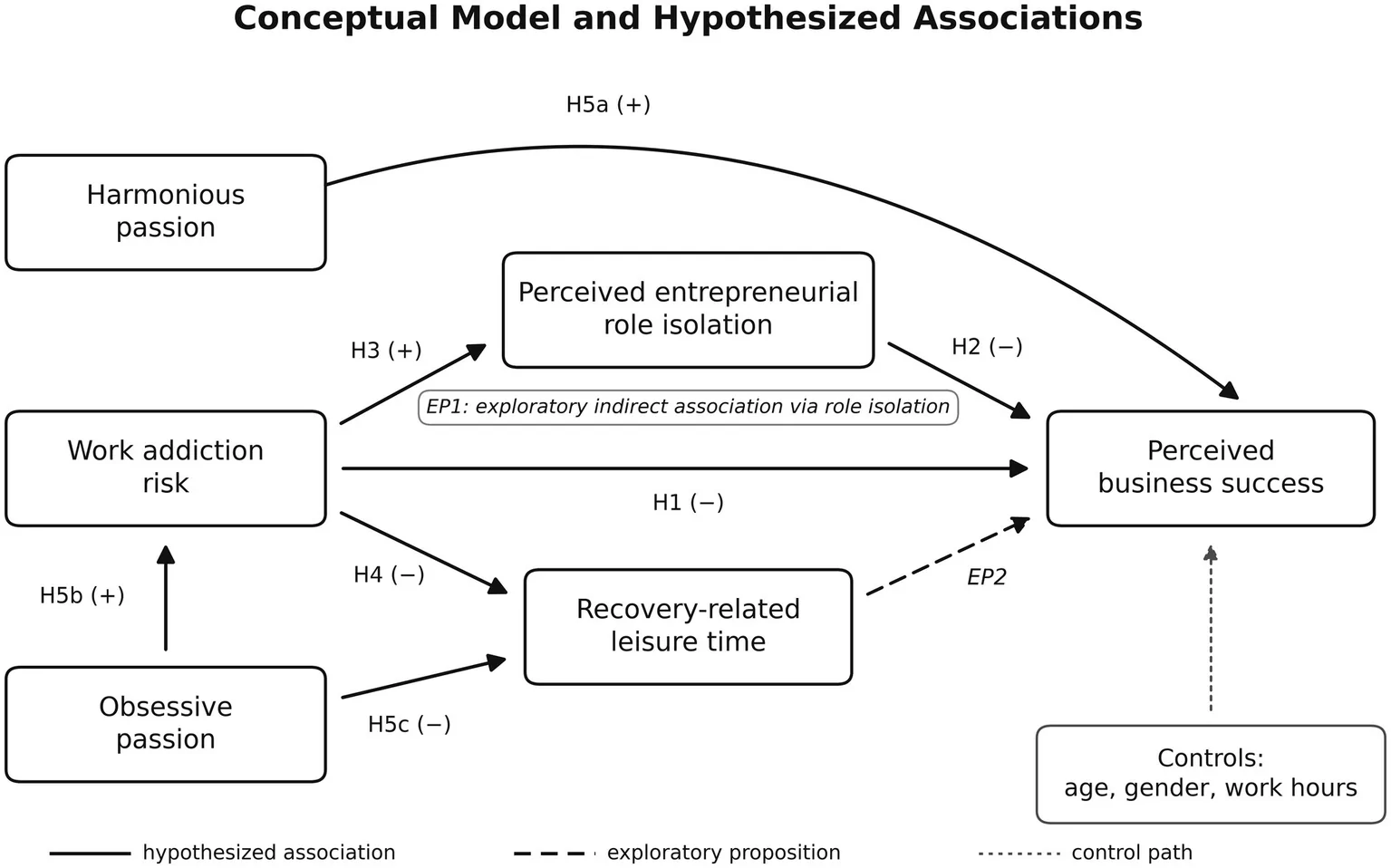
Conceptual model.

*EP1*. The negative association between work addiction risk and perceived business success will be attenuated when perceived entrepreneurial role isolation is added to the model, consistent with an indirect association through role isolation.

*EP2*. The negative association between work addiction risk and perceived business success will be attenuated when recovery-related leisure time is added to the model, consistent with an indirect association through recovery-related leisure time.

### 2.7 Research design and sample

The study employed a cross-sectional quantitative survey design to examine associations among work addiction risk, perceived entrepreneurial role isolation, recovery-related leisure time, work passion, and perceived business success among entrepreneurs and business leaders. Cross-sectional designs are appropriate for estimating theoretically specified associations, provided that temporal ordering and causal inference are not read into the data structure (Spector, 2019). All analyses were accordingly interpreted as associational rather than causal.

Data were collected in Hungary through an online cross-sectional survey administered on the LimeSurvey platform between May and November 2024. Participants were recruited through social media channels (Facebook, Instagram, LinkedIn, and Twitter, now X) and through the weekly newsletter of the National Association of Entrepreneurs and Employers, which circulated the call for participation among its members. The call was repeated weekly throughout the data collection period. Participation was voluntary and anonymous and was open to individuals aged 18 and older working in a managerial role, as stated in the call for participation and the consent form. Respondents provided informed consent electronically by selecting the yes option at the end of the online consent form. The Ethics Committee of the Széchenyi István University granted ethical permission for the study, SZE/ETT-3/2024 (V.26).

The initial respondent pool comprised 200 leaders: 151 men, 48 women, and one person who did not disclose gender. Sixteen respondents were excluded because their questionnaires were incomplete to a degree that precluded meaningful analysis. No other exclusion criteria were applied. The analytical dataset therefore comprised 184 respondents. Because the call circulated openly and no probability sampling frame existed, the sample is a self-selected convenience sample, and a conventional response rate cannot be computed. All respondents were Hungarian citizens. Most held a college degree or higher qualification (*n* = 141), and 43 respondents had completed secondary education. Respondents reported, on average, 19.68 years of managerial experience (SD = 9.73; available for 170 respondents). All of them held an ownership stake in the business they led (range 1–100%; Mdn = 66%), and 65 respondents were sole owners. Ownership was not an inclusion criterion; the composition of the achieved sample reflects the entrepreneur- and employer-focused recruitment channels. Most respondents occupied the top executive position (*n* = 124); the remainder identified as members of the executive leadership team (*n* = 27) or as co-owners (*n* = 33). They represented a broad range of industries, with manufacturing, mining, and energy forming the largest group (*n* = 71), and predominantly led firms in the maturity stage of the organizational life cycle (*n* = 121). The businesses operated in Budapest (*n* = 43), in county seats (*n* = 57), in other towns (*n* = 51), and in villages (*n* = 33).

Because all focal constructs were assessed through self-report, the design was treated as potentially vulnerable to common method bias. This limitation was addressed through conservative interpretation, robustness checks that included distress-related and firm-level covariates, and explicit avoidance of causal language (Podsakoff and Organ, 1986; Podsakoff et al., 2024).

### 2.8 Measures

*Perceived business success*. The dependent variable was perceived business success, operationalized as a composite index derived from three self-reported indicators. Perceived business performance ranged from heavily loss-making to highly profitable, revenue growth ranged from a decline of more than 25% to growth above 25%, and performance relative to competitors ranged from much worse to much better. Respondents who indicated on the competitor-relative item that they could not judge their standing were recoded as missing on that item before index construction. The main index was computed as the mean of at least two valid items, which retained cases with sufficient partial information. Two alternative operationalizations were used in robustness analyses: a complete three-item index and a two-item financial and business success index based on perceived business performance and revenue growth. This measurement strategy reflects the conceptual distinction between perceived economic and business success and full multidimensional subjective entrepreneurial success. The available indicators capture business-related self-evaluation rather than broader facets such as personal fulfilment, workplace relationships, community impact, or personal financial rewards. The use of a composite index was evaluated through internal consistency and item-level diagnostics, consistent with recommendations for scale and index construction (Boateng et al., 2018). The index was interpreted as perceived economic and business success. This interpretation is in line with entrepreneurship research that treats self-rated success as conceptually meaningful yet analytically distinct from objective indicators and broader wellbeing outcomes (Wiklund et al., 2019; Hamrick et al., 2023; Le Moal et al., 2026).

*Work addiction risk*. Work addiction risk was measured with the seven-item Bergen Work Addiction Scale (Andreassen et al., 2012). Items were rated on the standard five-point frequency scale, and the total score was computed as the sum of the seven items (possible range 7–35). The continuous score was used in the main analyses to preserve information and avoid unnecessary loss of variance. Consistent with the original scoring guideline (Andreassen et al., 2012), respondents who endorsed at least four of the seven items with a rating of 4 (often) or 5 (always) were classified as being at high risk of work addiction. This rule identified 56 respondents (30.4%) as high risk, coded 1; the remaining 128 respondents formed the control group, coded 0. The dual operationalization allowed the study to examine both dimensional associations and group-level differences.

*Perceived entrepreneurial role isolation*. Perceived entrepreneurial role isolation was measured with a single item asking how respondents had felt in their managerial role over the preceding months. The five-point scale was anchored from feeling very isolated and lonely to feeling very much surrounded, and responses were reverse-coded so that higher scores indicated greater perceived role isolation. Because this was a single-item measure, it was not treated as a validated loneliness or social isolation scale but as a role-specific proxy indicator of perceived isolation within the business leadership context. Single-item measures can be justified under specific conditions, but they require transparent conceptual restriction and explicit acknowledgement of their narrower measurement scope (Matthews et al., 2022).

*Recovery-related leisure time*. Recovery-related leisure time was measured as the number of daily leisure hours, excluding sleep, during which respondents reported not thinking about business-related tasks. The variable was treated as a proxy for detachment-related recovery capacity rather than as a validated psychological detachment scale. A winsorized version, capped at 8 h, was created to reduce the influence of extreme upper-end values in robustness analyses.

*Work passion*. Harmonious and obsessive passion were measured with six items per subscale, grounded in the dualistic model of passion (Vallerand et al., 2003). Items were administered on a seven-point agreement scale, recoded to a 0–6 metric, and summed; both subscale scores therefore have a structural range of 0–36. Five additional criterion items assessing general passion for work were administered but were not used in the present analyses. Harmonious passion represented more autonomous and integrated work involvement, whereas obsessive passion represented more controlled and rigid work-related motivation. The two variables were used to examine whether adaptive and maladaptive motivational profiles showed distinct associations with perceived business success, work addiction risk, perceived role isolation, and recovery-related leisure time.

*Firm-level covariates*. Robustness analyses additionally drew on firm-level covariates. Firm size was operationalized as the natural logarithm of the number of employees plus one, and ownership share was recorded as a percentage. Firm life-cycle stage was coded as early, maturity, and stabilization or decline, with maturity as the reference category; the start-up category comprised a single case and was therefore merged with early growth. Leadership position distinguished the top executive (reference), members of the executive leadership team, and co-owners, and industry comprised eight categories. These variables were self-reported and categorical in nature. They were used to probe the robustness of the focal associations rather than as substantive predictors.

### 2.9 Data preparation and statistical analysis

Data preparation involved recoding the competitor-relative performance item, reversing the role-isolation item, constructing the perceived business success indices, coding high work addiction status, and preparing gender for regression models. Descriptive statistics were computed for all focal variables. Distributional characteristics and potential outliers were inspected, with particular attention to recovery-related leisure time and work hours. The screening criterion for implausible responses was structural: values at or beyond the physical upper bound of the response scale were flagged. Weekly working hours equal to the total number of hours in a week (168; *n* = 4) and recovery-related leisure time of 20 h per day, which approximates the entire waking day (*n* = 2), met this criterion; the six flagged responses came from six different respondents. The survey contained no formal attention-check items, so no further behavioural screening was available. The flagged cases were retained in the main analyses to avoid discretionary exclusions and because the rank-based bivariate procedures are insensitive to extreme values. A winsorized recovery variable and sensitivity analyses excluding the flagged cases are reported in the robustness section. Missing values were handled using pairwise deletion for bivariate correlations and listwise deletion for regression models. This approach retained maximum information for preliminary associations while maintaining consistent case bases within multivariate models.

Internal consistency was assessed for multi-item scales and indices using Cronbach’s alpha, interpreted strictly as an indicator of internal consistency rather than construct validity (Tavakol and Dennick, 2011). Internal consistency was not calculated for perceived role isolation or recovery-related leisure time, which were single-item proxy variables. Bivariate associations were examined using Spearman rank-order correlations, appropriate because several measures were ordinal, single-item, or non-normally distributed. Pearson correlations were treated only as sensitivity checks where distributional assumptions were reasonable (Bishara and Hittner, 2012; Bonett and Wright, 2000). Where the magnitudes of two dependent correlations are compared, the difference was tested with Steiger’s *z* on the rank-transformed data and with a bootstrapped confidence interval for the difference. Group comparisons between respondents with and without high work addiction risk used Mann–Whitney *U* tests as the primary nonparametric procedure, accompanied by group means, standard deviations, and effect size estimates.

The primary multivariate analysis used hierarchical linear regression with perceived business success as the dependent variable. Age, gender, and weekly work hours were entered as controls. Work addiction risk was added in the second block, perceived role isolation and recovery-related leisure time in the third, and harmonious and obsessive passion in the final main model. All hierarchical models were estimated on the constant listwise sample (*n* = 182), so that the blocks are strictly nested, and the significance of each block increment was evaluated with F tests for the change in *R*^2^. Model fit was evaluated using *R*^2^ and changes in *R*^2^ across blocks, and regression coefficients, standard errors, confidence intervals, significance levels, and multicollinearity diagnostics were examined. Robustness analyses took several forms. The hierarchical models were repeated with alternative success outcomes, namely the complete three-item index, the two-item financial and business index, and each individual success item, and with the winsorized recovery variable substituted for the raw measure. Further models added distress-related controls (perceived stress, burnout exhaustion, or perceived mental health) or the firm-level covariates described above. The final model was also re-estimated after excluding the flagged time-use reports and, separately, within the top-executive subsample. Supplementary logistic regression models examined predictors of high work addiction risk; given the modest number of high-risk cases, the number of predictors was restricted to limit overfitting, consistent with evidence on events-per-variable requirements (Vittinghoff and McCulloch, 2007). Exploratory indirect-association analyses used bootstrapped estimates (5,000 resamples) of the indirect path from work addiction risk to perceived business success through role isolation and through recovery-related leisure time (Preacher and Hayes, 2008). Given the cross-sectional design, these were interpreted as statistical decompositions of association, not as causal mediation (Maxwell and Cole, 2007; O’Laughlin et al., 2018).

## 3 Results

### 3.1 Sample characteristics and measurement diagnostics

The analytic sample comprised 184 entrepreneurs and business leaders. Respondents were, on average, 54.30 years old (SD = 10.76); 140 were male, 43 female, and one respondent did not report gender. In terms of work addiction classification, 56 respondents (30.4%) met the high-risk criterion, and 128 formed the control group.

The perceived business success index showed acceptable internal consistency, Cronbach’s *α* = 0.726, based on complete-item cases, as did the Bergen Work Addiction Scale, *α* = 0.779. Reliability estimates for harmonious passion, *α* = 0.809, and obsessive passion, *α* = 0.772, were likewise satisfactory. Internal consistency was not calculated for perceived role isolation or recovery-related leisure time, which were single-item proxy variables. Descriptive statistics (Table 1) showed sufficient variability across all focal constructs. Perceived business success had a mean of 3.20 (SD = 0.71, median = 3.33, range 1–5). The mean work addiction score was 20.01 (SD = 4.63), perceived role isolation averaged 3.01 (SD = 0.93), and recovery-related leisure time averaged 3.26 h per day (SD = 3.07, median = 3.00, range 0–20). Because the recovery variable showed substantial dispersion and potential upper-end outliers, the winsorized version capped at 8 h was used in robustness analyses. Harmonious passion had a mean of 24.09 (SD = 6.07) and obsessive passion of 11.75 (SD = 6.29). Given the identical scoring of the two subscales, both scores range structurally from 0 to 36, and the reported minima and maxima are observed values.

**Table 1:** Descriptive statistics and internal consistencies.

Variable	*n*	M	SD	Median	Range
Perceived business success	184	3.20	0.71	3.33	1–5
Work addiction risk	184	20.01	4.63	20.00	9–30
Role isolation	184	3.01	0.93	3.00	1–5
Recovery-related leisure time	183	3.26	3.07	3.00	0–20
Harmonious passion	184	24.09	6.07	25.00	8–36
Obsessive passion	184	11.75	6.29	11.50	0–31
Weekly work hours	184	48.78	24.89	50.00	1–168
Perceived stress	184	5.14	2.75	5.00	0–15
Burnout exhaustion	184	11.76	3.23	12.00	5–20
Perceived mental health	176	3.66	0.78	4.00	2–5
Sleep quality	176	3.39	0.92	3.00	2–5

Role isolation and recovery-related leisure time are single-item/proxy variables. Ranges are observed values; the harmonious and obsessive passion subscales share a structural range of 0–36.

### 3.2 Bivariate associations

Spearman rank-order correlations provided initial support for several hypotheses (Table 2). Work addiction risk was negatively associated with perceived business success, *ρ* = −0.25, *p* < 0.001, supporting H1 at the bivariate level. The modest magnitude indicates that work addiction risk alone does not provide a comprehensive account of perceived business success. Perceived role isolation was also negatively associated with perceived business success, *ρ* = −0.34, *p* < 0.001, supporting H2. The isolation correlation was numerically larger than the work addiction correlation, but the difference between the two dependent correlations was not significant (Steiger *z* = −1.28, *p* = 0.199; bootstrapped difference = −0.09, 95% CI [−0.24, 0.05]), so the two bivariate associations should be regarded as comparable in magnitude. Work addiction risk was strongly and positively associated with role isolation, *ρ* = 0.48, *p* < 0.001, supporting H3, and negatively associated with recovery-related leisure time, *ρ* = −0.41, *p* < 0.001, supporting H4 at the bivariate level. Recovery-related leisure time, however, was not meaningfully associated with perceived business success, *ρ* = 0.07, *p* = 0.337. This early pattern suggests that recovery characterizes the work addiction profile without translating directly into business self-evaluation.

**Table 2:** Key Spearman correlations for hypotheses.

Association	*n*	*ρ*	*p*
Work addiction risk and perceived business success	184	−0.25	< 0.001
Role isolation and perceived business success	184	−0.34	< 0.001
Work addiction risk and role isolation	184	0.48	< 0.001
Work addiction risk and recovery-related leisure time	183	−0.41	< 0.001
Harmonious passion and perceived business success	184	0.28	< 0.001
Obsessive passion and perceived business success	184	−0.03	0.690
Obsessive passion and work addiction risk	184	0.51	< 0.001
Obsessive passion and recovery-related leisure time	183	−0.25	< 0.001
Recovery-related leisure time and perceived business success	183	0.07	0.337

Two-tailed tests.

The passion variables showed differentiated patterns. Harmonious passion was positively associated with perceived business success, *ρ* = 0.28, *p* < 0.001, supporting H5a at the bivariate level, and it was negatively associated with work addiction risk and role isolation. Obsessive passion was strongly and positively associated with work addiction risk, *ρ* = 0.51, *p* < 0.001, supporting H5b, and negatively associated with recovery-related leisure time, *ρ* = −0.25, *p* < 0.001, supporting H5c. Obsessive passion was not significantly associated with perceived business success, *ρ* = −0.03, *p* = 0.690. Harmonious and obsessive passion thus differentiate more adaptive and more maladaptive work-investment profiles, but their direct associations with perceived business success are not equally strong.

### 3.3 Group comparisons by work addiction risk

Mann–Whitney *U* tests compared respondents with and without high work addiction risk (Table 3). The high-risk group reported lower perceived business success (*M* = 2.98, SD = 0.75) than the control group (*M* = 3.30, SD = 0.68), *U* = 2655.00, *p* = 0.005, Cohen’s *d* = −0.46, which further supports H1 with a moderate group difference. The largest difference concerned perceived role isolation. The high-risk group reported substantially higher isolation (*M* = 3.55, SD = 0.91) than the control group (*M* = 2.77, SD = 0.83), *U* = 5241.00, *p* < 0.001, *d* = 0.92, reinforcing H3. The high-risk group also reported less recovery-related leisure time (*M* = 2.41, SD = 3.43, vs. *M* = 3.64, SD = 2.83), *U* = 2050.00, *p* < 0.001, *d* = −0.41, supporting H4 at the group level.

**Table 3:** Group comparisons by high work addiction risk.

Variable	Control M (SD)	High-risk M (SD)	*U*	*p*	*d*
Perceived business success	3.30 (0.68)	2.98 (0.75)	2655.0	0.005	−0.46
Role isolation	2.77 (0.83)	3.55 (0.91)	5241.0	<0.001	0.92
Recovery-related leisure time	3.64 (2.83)	2.41 (3.43)	2050.0	<0.001	−0.41
Harmonious passion	25.16 (5.83)	21.66 (5.97)	2417.5	<0.001	−0.60
Obsessive passion	10.13 (5.71)	15.45 (6.03)	5292.0	<0.001	0.91
Perceived stress	4.41 (2.48)	6.79 (2.62)	5308.5	<0.001	0.94
Burnout exhaustion	10.76 (2.88)	14.05 (2.79)	5719.0	<0.001	1.15
Perceived mental health	3.78 (0.68)	3.37 (0.92)	2270.0	0.001	−0.53
Sleep quality	3.57 (0.86)	2.96 (0.92)	2029.0	<0.001	−0.69

High-risk group coded as 1; control group coded as 0. *U* = Mann–Whitney *U*. Cohen’s d reflects high-risk minus control.

The passion variables likewise differentiated the groups, consistent with H5b and H5c. The high-risk group reported lower harmonious passion (*M* = 21.66, SD = 5.97, vs. *M* = 25.16, SD = 5.83), *U* = 2417.50, *p* < 0.001, *d* = −0.60, and higher obsessive passion (*M* = 15.45, SD = 6.03, vs. *M* = 10.13, SD = 5.71), *U* = 5292.00, *p* < 0.001, *d* = 0.91. The high-risk group also showed a broader adverse psychological profile, with higher perceived stress, higher burnout exhaustion, lower perceived mental health, and poorer sleep quality. High work addiction risk is thus embedded in a wider unfavourable profile of work functioning and strain.

### 3.4 Hierarchical regression predicting perceived business success

Hierarchical linear regression tested whether the focal variables explained perceived business success beyond demographic and work-related controls (Tables 4, 5). All models were estimated on the constant listwise sample of 182 respondents, so that each block increment could be tested with an F test for the change in *R*^2^. The control model, including age, gender, and weekly work hours, did not explain a meaningful proportion of variance, *R*^2^ = 0.007, adjusted *R*^2^ = −0.010, *F* = 0.41, *p* = 0.744. Adding work addiction risk significantly improved the model, *R*^2^ = 0.079, Δ*R*^2^ = 0.072, *F* change (1, 177) = 13.89, *p* < 0.001. Work addiction risk was negatively associated with perceived business success, *β* = −0.27, *p* < 0.001, supporting H1 in the control-adjusted model.

**Table 4:** Standardized coefficients across hierarchical regression models.

Predictor	M1	M2	M3	M4
Age	−0.07	−0.13†	−0.13^†^	−0.14^*^
Female	−0.04	−0.02	0.01	0.01
Weekly work hours	−0.03	−0.01	−0.01	−0.01
Work addiction risk		−0.27^***^	−0.13	−0.15
Role isolation			−0.33^***^	−0.28^***^
Recovery-related leisure time			−0.02	−0.02
Harmonious passion				0.14^†^
Obsessive passion				0.08

Values are standardized *β* coefficients; all models estimated on the constant listwise sample (*n* = 182). ^†^*p* < 0.10, ^*^p < 0.05, ^**^p < 0.01, ^***^p < 0.001 (two-tailed).

**Table 5:** Hierarchical regression model summary predicting perceived business success.

Model	*R* ^2^	Adj. *R*^2^	*F*	*p*	Δ*R*^2^	*F* change (*df*)	*p* change
M1	0.007	−0.010	0.41	0.744			
M2	0.079	0.058	3.80	0.005	0.072	13.89 (1, 177)	<0.001
M3	0.161	0.133	5.61	<0.001	0.082	8.57 (2, 175)	<0.001
M4	0.188	0.150	4.99	<0.001	0.026	2.80 (2, 173)	0.064

All models estimated on the constant listwise sample (*n* = 182). M1 = controls; M2 = + work addiction; M3 = + role isolation and recovery-related leisure time; M4 = + harmonious and obsessive passion. *F* change tests the increment of each block over the preceding model.

The interpretation changed once perceived role isolation and recovery-related leisure time were entered. This block further improved fit, *R*^2^ = 0.161, Δ*R*^2^ = 0.082, *F* change (2, 175) = 8.57, *p* < 0.001. Role isolation emerged as a strong negative predictor, recovery-related leisure time was not significant, and the work addiction coefficient was attenuated. The bivariate association between work addiction and perceived success is therefore not best understood as a stable direct association. The final block, adding harmonious and obsessive passion, produced only a marginal improvement in fit, Δ*R*^2^ = 0.026, *F* change (2, 173) = 2.80, *p* = 0.064, and the final model explained 18.8% of the variance, adjusted *R*^2^ = 0.150. In the final model, role isolation remained the most consistent focal predictor, *β* = −0.28, *p* < 0.001. Work addiction risk, *β* = −0.15, *p* = 0.149, and recovery-related leisure time remained non-significant, harmonious passion showed only a weak positive association, and obsessive passion showed no significant direct association.

Two qualifications apply. Because role isolation was measured with a single item and work addiction risk with a multi-item scale, differential reliability attenuation complicates direct comparisons of coefficient magnitudes. The claim of relative stability therefore rests on the consistency of the isolation coefficient across specifications rather than on its size relative to other predictors; a bootstrap comparison of the two coefficients in the final model likewise did not indicate a significant difference (*β* difference = −0.14, 95% CI [−0.41, 0.17]). Age, in turn, showed a modest negative association in the final model, *β* = −0.14, *p* < 0.05, indicating slightly less favourable business self-evaluations among older respondents; this association was not hypothesized and is not interpreted further.

These findings support H2 most strongly. H1 is supported bivariately and in the control-adjusted model, but not in the fully adjusted model. H4 is supported in relation to work addiction risk but not as a predictor of perceived business success. The passion hypotheses receive differentiated support: H5b and H5c are supported at the bivariate and group levels, whereas H5a is supported bivariately with only a marginal association in the final model.

### 3.5 Robustness analyses

Robustness analyses tested at the outset whether the main findings depended on how perceived business success was operationalized (Table 6). Role isolation remained negatively associated with the complete three-item index and the two-item financial and business index. It also significantly predicted self-rated business performance and revenue growth when these indicators were analysed separately; the association with competitor-relative performance was weaker and did not reach conventional significance. Work addiction risk did not remain significant in any fully adjusted robustness model. Its standardized coefficients were consistently negative but non-significant once role isolation and motivational variables were included. Recovery-related leisure time was not a significant predictor in any perceived success model. This pattern was unchanged when the winsorized version was used, which indicates that the absence of a direct recovery and success relationship is not attributable to extreme values.

**Table 6:** Robustness checks across alternative perceived success outcomes.

Outcome	*R* ^2^	WA risk	Role isolation	Recovery	HP	OP
PBS, complete 3-item index	0.184	−0.15 (0.159)	−0.30^***^ (<0.001)	−0.02 (0.785)	0.12 (0.152)	0.07 (0.464)
Two-item financial success	0.168	−0.13 (0.208)	−0.30^***^ (<0.001)	−0.04 (0.616)	0.09 (0.278)	0.03 (0.723)
Business performance	0.164	−0.10 (0.328)	−0.25^**^ (0.003)	0.02 (0.765)	0.15^†^ (0.070)	−0.03 (0.729)
Revenue growth	0.135	−0.13 (0.213)	−0.29^***^ (<0.001)	−0.08 (0.279)	0.02 (0.803)	0.08 (0.379)
Competitor-relative performance	0.108	−0.12 (0.259)	−0.14 (0.108)	0.03 (0.696)	0.16^†^ (0.065)	0.13 (0.187)
PBS with winsorized recovery time	0.189	−0.15 (0.134)	−0.28^***^ (<0.001)	−0.04 (0.610)	0.14^†^ (0.089)	0.08 (0.395)

Values are standardized *β* coefficients with *p* values in parentheses. Same predictor structure as the final hierarchical model. PBS = perceived business success; WA = work addiction; HP = harmonious passion; OP = obsessive passion. ^†^*p* < 0.10, ^*^p < 0.05, ^**^p < 0.01, ^***^p < 0.001.

Additional robustness models included perceived stress, burnout exhaustion, or perceived mental health as distress-related controls (Table 7). Perceived role isolation remained significant across these models, whereas work addiction risk remained non-significant. The role-isolation finding is therefore unlikely to reflect general distress or negative self-evaluation alone.

**Table 7:** Distress-control robustness models predicting perceived business success.

Control added	*R* ^2^	WA risk	Role isolation	Recovery	Added control
Perceived stress	0.207	−0.12 (0.217)	−0.22^**^ (0.010)	−0.02 (0.820)	−0.20^*^ (0.040)
Burnout exhaustion	0.188	−0.13 (0.210)	−0.28^***^ (< 0.001)	−0.02 (0.754)	−0.04 (0.681)
Perceived mental health	0.182	−0.17 (0.108)	−0.28^**^ (0.001)	0.00 (0.957)	−0.06 (0.511)

Each model adds one distress-related control to the final hierarchical model. Values are *β* coefficients with *p* values in parentheses. ^†^*p* < 0.10, ^*^p < 0.05, ^**^p < 0.01, ^***^p < 0.001.

A further set of robustness models examined whether the focal associations were attributable to firm-level heterogeneity (Table 8). This check was warranted because perceived role isolation was negatively correlated with firm size (*ρ* = −0.19, *p* = 0.010). Smaller firms could therefore, in principle, account for both greater isolation and less favourable business self-evaluations. Adding firm size and ownership share to the final hierarchical model left the role-isolation coefficient essentially unchanged (*β* = −0.24, *p* = 0.004), while firm size itself was positively associated with perceived business success (*β* = 0.23, *p* = 0.003). Adding life-cycle stage and leadership position, and subsequently industry categories, did not alter this pattern. Perceived role isolation remained a significant negative correlate across all specifications (β between −0.21 and −0.24, all *p* < 0.01). Firms in the stabilization or decline stage reported substantially lower perceived business success (*β* = −0.39, *p* < 0.001), a pattern consistent with the face validity of the outcome measure. Work addiction risk reached conventional significance in one specification (*β* = −0.18, *p* = 0.047) but was not consistently significant across specifications, mirroring the pattern observed in the main models.

**Table 8:** Robustness of the final model to firm-level covariates, data-screening exclusions, and subsample restriction.

Specification	*n*	*R* ^2^	WA risk	Role isolation	Recovery	HP	OP
Model A: firm size and ownership share added	182	0.237	−0.15 (0.124)	−0.24 (0.004)	−0.02 (0.770)	0.15 (0.069)	0.08 (0.348)
Model B: Model A + life-cycle stage and leadership position	182	0.381	−0.18 (0.047)	−0.21 (0.006)	0.00 (0.964)	0.12 (0.111)	0.06 (0.455)
Model C: Model B + industry	182	0.433	−0.15 (0.096)	−0.23 (0.003)	0.01 (0.938)	0.12 (0.094)	0.05 (0.568)
Model D: exclusion of flagged time-use reports	176	0.195	−0.18 (0.087)	−0.27 (0.001)	−0.01 (0.914)	0.14 (0.099)	0.08 (0.392)
Model E: top-executive subsample	123	0.152	0.00 (0.998)	−0.31 (0.002)	−0.05 (0.569)	0.15 (0.138)	−0.03 (0.771)

Values are standardized *β* coefficients with *p* values in parentheses; perceived business success is the dependent variable. All models include age, gender, and weekly work hours as covariates, alongside the predictors shown. Model A adds ln(number of employees + 1) and ownership share to the final hierarchical model; Model B adds life-cycle stage (reference: maturity) and leadership position (reference: top executive); Model C adds industry (eight categories). Model D re-estimates the final hierarchical model after excluding the flagged time-use reports; Model E re-estimates it within the top-executive subsample. WA = work addiction; HP = harmonious passion; OP = obsessive passion.

Two further checks addressed data quality and sample composition. Excluding respondents with the flagged time-use reports (weekly working hours of 168, *n* = 4; recovery-related leisure time of 20 h per day, *n* = 2) did not change the conclusions. Perceived role isolation remained significantly negatively associated with perceived business success (*β* = −0.27, *p* = 0.001), and work addiction risk remained non-significant. A separate check restricted the sample to respondents holding the top executive position (*n* = 123). This subsample yielded a comparable, if anything stronger, role-isolation coefficient (*β* = −0.31, *p* = 0.002), whereas the residual association of work addiction risk was close to zero. This exploratory subsample analysis suggests that the role-isolation finding is not driven by heterogeneity in leadership position, although the modest subsample size warrants caution.

### 3.6 Supplementary logistic regression predicting high work addiction risk

Supplementary logistic regression examined whether perceived role isolation, recovery-related leisure time, harmonious passion, and obsessive passion predicted high work addiction risk (Table 9). The baseline model including age, gender, and weekly work hours had limited explanatory value, Nagelkerke *R*^2^ = 0.040, AUC = 0.609, classification accuracy = 69.9%, with no significant control-variable predictors. Adding role isolation and recovery-related leisure time substantially improved the model. Role isolation positively predicted high work addiction risk, whereas recovery-related leisure time was not significant after controls. The final model, adding harmonious and obsessive passion, showed stronger explanatory and classification performance, Nagelkerke *R*^2^ = 0.421, AUC = 0.839, classification accuracy = 80.2%. Classification performance was asymmetric (specificity = 89.7%, sensitivity = 58.9%), indicating that the model identified control-group members more reliably than high-risk respondents.

**Table 9:** Supplementary logistic regression predicting high work addiction risk.

Predictor	OR	95% CI	*p*
Age	1.00	0.96–1.04	0.910
Female	0.88	0.35–2.24	0.789
Weekly work hours	1.00	0.99–1.02	0.689
Role isolation	2.42	1.40–4.16	0.001
Recovery-related leisure time	0.93	0.80–1.08	0.337
Harmonious passion	0.90	0.84–0.97	0.005
Obsessive passion	1.19	1.10–1.29	<0.001

Final model: *n* = 182, AUC = 0.84, accuracy = 80.2%, sensitivity = 58.9%, specificity = 89.7%.

In the final model, role isolation was positively associated with high work addiction risk, OR = 2.42, 95% CI [1.40, 4.16], *p* = 0.001. Harmonious passion was negatively associated, OR = 0.90, 95% CI [0.84, 0.97], *p* = 0.005, and obsessive passion was positively associated, OR = 1.19, 95% CI [1.10, 1.29], *p* < 0.001, a pattern consistent with H5b, while recovery-related leisure time did not remain significant, OR = 0.93, *p* = 0.337. High work addiction risk is thus characterized less by recovery-related leisure time alone than by a broader motivational and role-functioning profile involving higher role isolation, lower harmonious passion, and higher obsessive passion.

### 3.7 Exploratory indirect association analyses

Exploratory analyses examined whether the association between work addiction risk and perceived business success was statistically consistent with an indirect pathway through perceived role isolation or recovery-related leisure time. Given the cross-sectional data, these analyses are interpreted as exploratory associations, not causal mediation. The isolation pathway was supported. Work addiction risk was positively associated with role isolation, and role isolation was negatively associated with perceived business success when work addiction risk and controls were included. The bootstrapped indirect association was negative, with a 95% confidence interval excluding zero, estimate = −0.023, 95% CI [−0.037, −0.011], and the attenuation of the work addiction coefficient documented in Section 4.4 is consistent with this pattern. EP1 was therefore supported as an exploratory, non-causal indirect association. The recovery pathway was not supported. Although work addiction risk was negatively associated with recovery-related leisure time, recovery-related leisure time was not associated with perceived business success under the same specification. The bootstrapped indirect association included zero, estimate = −0.000, 95% CI [−0.007, 0.006]. EP2 was therefore not supported. The empirical pattern is thus more consistent with an isolation-based than with a recovery-based account of the association between work addiction and perceived success (Table 10).

**Table 10:** Exploratory bootstrapped indirect associations.

Mediator	*n*	*a*	*b*	Indirect	95% boot CI
Role isolation	183	0.093	−0.251	−0.023	[−0.037, −0.011]
Recovery-related leisure time	182	−0.178	0.002	−0.000	[−0.007, 0.006]

Unstandardized estimates. Bootstrap samples = 5,000. Indirect associations are not interpreted as causal mediation.

### 3.8 Summary of hypothesis testing

The results provide mixed but theoretically informative support for the proposed model (Table 11; Figure 2). H1 was partially supported: work addiction risk was negatively associated with perceived business success in bivariate analyses, group comparisons, and the control-adjusted regression, but the association became non-significant in the final hierarchical model. H2 was supported: perceived role isolation was consistently negatively associated with perceived business success across bivariate, multivariate, and robustness analyses, including models with distress-related and firm-level covariates. H3 was supported: work addiction risk was strongly associated with higher role isolation. H4 was supported at the bivariate and group-comparison levels, although recovery-related leisure time did not independently predict perceived business success. H5a was supported at the bivariate level, with only a marginal association in the final model. H5b was supported, and H5c was supported at the bivariate and group-comparison levels. EP1 was supported as an exploratory indirect association; EP2 was not.

**Table 11:** Hypothesis support summary.

Hypothesis	Result	Evidence
H1 WA risk → PBS	Partial	Bivariate negative; non-significant in final model.
H2 Isolation → PBS	Supported	Consistent negative predictor across main and robustness models, including firm-level covariates.
H3 WA risk → Isolation	Supported	Strong positive association.
H4 WA risk → Recovery	Bivariate only	Correlation/group difference supported; not independent in models.
H5a HP → PBS	Partial	Positive bivariate association; marginal in final model.
H5b OP → WA risk	Supported	Strong positive association in correlational and logistic analyses.
H5c OP → Recovery	Bivariate only	Negative bivariate and group-level association.
EP1 Isolation path	Supported exploratory	Attenuation of the focal association; bootstrap CI did not include zero.
EP2 Recovery path	Not supported	No attenuation through recovery; bootstrap CI included zero.

Support labels reflect the combined pattern across bivariate, regression, robustness, and supplementary analyses. Arrows denote hypothesized associations, not causal directions. #5. Discussion and Conclusion.

**Figure 2:**
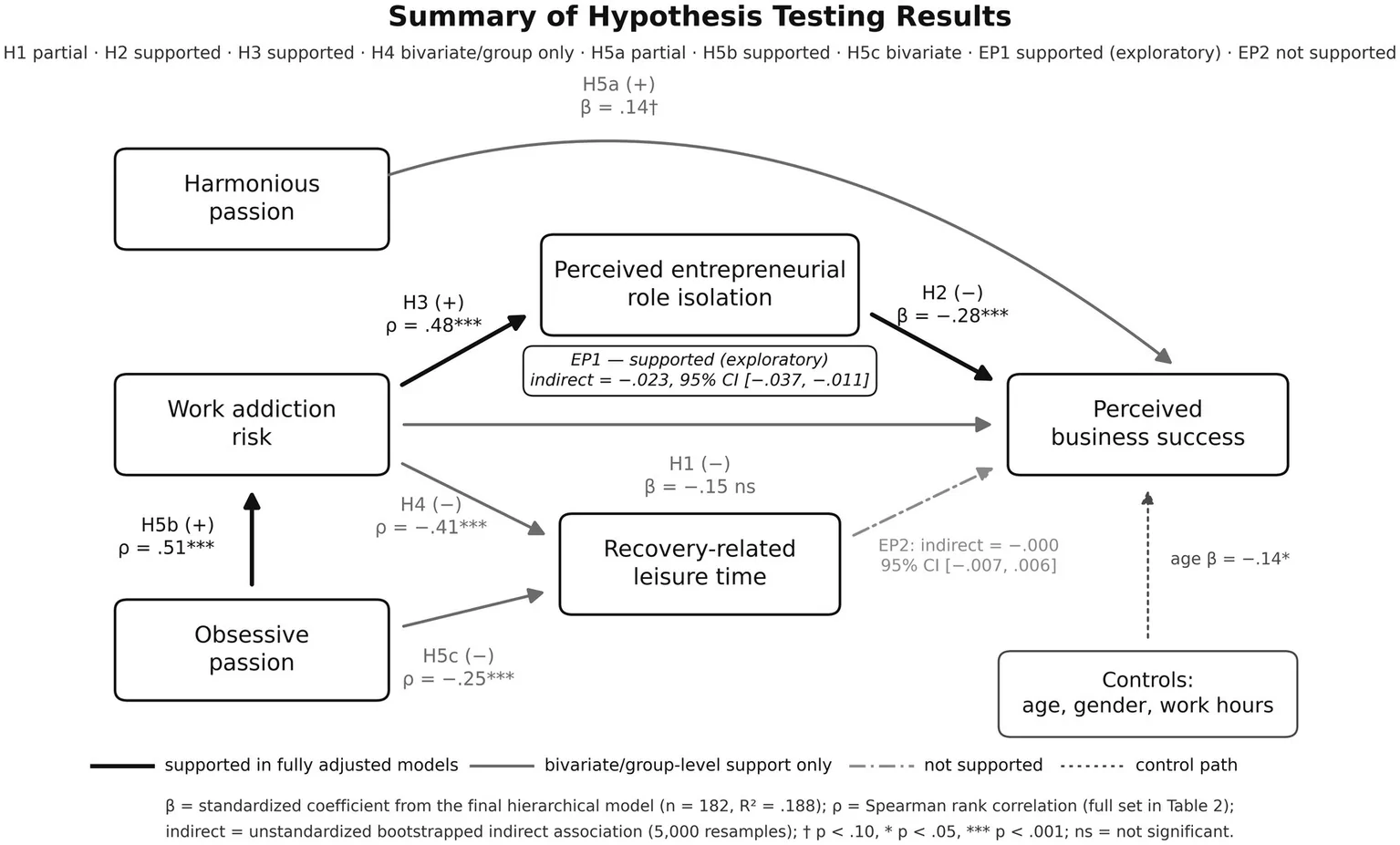
Summary of hypothesis testing.

The central empirical claim should be stated with precision. The strongest result is not a stable direct association between work addiction risk and perceived business success. It is the consistency with which perceived entrepreneurial role isolation was negatively associated with perceived business success across specifications. Work addiction risk remains relevant because it is embedded in a broader maladaptive profile involving higher role isolation, lower recovery-related leisure time, lower harmonious passion, and higher obsessive passion. Recovery-related leisure time characterizes this profile without independently explaining perceived business success in the present data.

### 3.9 Summary of key findings

This study asked how work addiction risk, perceived entrepreneurial role isolation, recovery-related leisure time, and work passion are associated with perceived business success among entrepreneurs and business leaders. The answer is more differentiated than a simple account in which work addiction depresses perceived success. Work addiction risk was negatively associated with perceived business success at the bivariate level, and respondents at high risk reported lower perceived business success than the control group. This association nonetheless became non-significant once role isolation and motivational variables entered the hierarchical models. The most consistent empirical result was instead the negative association between perceived role isolation and perceived business success. It held in bivariate analyses, in the final hierarchical model, and across every robustness specification examined. These specifications included alternative success indices, distress-related controls, and firm-level covariates for firm size, ownership share, life-cycle stage, leadership position, and industry. They also included the exclusion of the flagged time-use reports and the restriction to the top-executive subsample. Because role isolation was measured with a single item, claims about the relative magnitude of its coefficient against multi-item predictors must remain guarded. The weight of the finding rests on its consistency across specifications, not on coefficient comparisons across measures of unequal reliability.

Two further results complete the picture. Recovery-related leisure time was clearly implicated in the work addiction profile. It was lower among high-risk respondents and negatively correlated with work addiction risk, yet it was not associated with perceived business success in any bivariate or multivariate analysis. The exploratory indirect pathway through recovery was accordingly not supported, whereas the pathway through role isolation was. The passion variables behaved as the dualistic model would lead one to expect (Vallerand et al., 2003). Obsessive passion was strongly associated with work addiction risk and lower recovery-related leisure time. Harmonious passion was associated with lower work addiction risk and, bivariately, with more favourable business self-evaluation. Neither directly explained perceived business success once role isolation was in the model. Work addiction risk thus matters, but primarily as part of a broader maladaptive role-functioning profile rather than as an independent direct correlate of business self-evaluation. The profile involves greater isolation, impaired recovery, lower harmonious and higher obsessive passion, and elevated strain.

### 3.10 Contribution to perceived business success research

The findings contribute to perceived entrepreneurial success research in two principal ways, within a deliberately bounded outcome definition. The study measured perceived economic and business success through self-rated performance, revenue development, and competitor-relative performance rather than full multidimensional subjective entrepreneurial success. This scope decision, made explicit at the design stage, prevents overextension of the findings toward facets such as personal fulfilment, community impact, or workplace relationships that the indicators do not capture (Wach et al., 2016, 2020).

One contribution concerns maladaptive work investment. Prior research established that entrepreneurial success evaluations are multidimensional and psychologically embedded. Wach et al. (2016, 2020) showed that entrepreneurs evaluate success against personally meaningful criteria, and Dijkhuizen et al. (2018) showed that wellbeing predicts later subjective success more clearly than objective performance. The present study extends this literature by testing whether maladaptive work investment, indexed here by work addiction risk, relates to business self-evaluation. The answer qualifies rather than confirms a direct relationship: the association is real at the bivariate level but does not survive adjustment for role isolation and motivational quality. This result complements Gorgievski et al. (2014), who found that workaholism relates to entrepreneurial outcomes through affective pathways, and it cautions against treating high work investment as either uniformly beneficial or uniformly harmful.

The other contribution concerns the social experience of the leadership role. Prior work linked success evaluations to values, wellbeing, and psychological functioning (Gorgievski et al., 2011; Dijkhuizen et al., 2018; Shir et al., 2019; Stephan, 2018). The present findings add perceived role isolation as a robust correlate of perceived business success, one that was not accounted for by the distress-related, motivational, and firm-level covariates available in this study. That the association persisted when firm size, ownership share, life-cycle stage, leadership position, and industry were controlled is consequential, because smaller firms could plausibly have produced both greater isolation and less favourable self-evaluations. The data give no indication that the isolation and success association is merely a small-firm artefact. How entrepreneurs and business leaders evaluate the economic condition of their business appears to depend not only on what the business does and how motivated the leader is, but also on how socially embedded or isolated the leader feels in the role. This implication is consistent with evidence that success perceptions are embedded in social-relational judgments (Aguinis et al., 2008).

### 3.11 Contribution to work addiction and entrepreneurial functioning research

The study also contributes to work addiction research by examining work addiction risk in relation to perceived business success rather than only wellbeing, burnout, or work-life conflict. The results are consistent with the adverse-profile view established meta-analytically (Clark et al., 2016). Respondents at high risk of work addiction reported higher role isolation, lower recovery-related leisure time, lower harmonious and higher obsessive passion, higher stress, higher burnout exhaustion, poorer mental health, and poorer sleep quality. The results caution at the same time against overstating work addiction risk as a direct predictor of business success. Its negative association weakened once role isolation and passion variables were included, and it was essentially absent within the top-executive subsample. This pattern supports the agenda of understanding entrepreneurial wellbeing through the distinctive psychological and contextual features of entrepreneurship rather than through employee-based assumptions alone (Wiklund et al., 2019; Stephan, 2018).

The findings also speak to recovery research in entrepreneurial contexts (Le Moal et al., 2026; Wach et al., 2021). Recovery-related leisure time was clearly tied to work addiction risk but not to perceived business success. Recovery may therefore be more proximal to mental health, strain, fatigue, and sleep than to economic and business self-evaluation. Recovery remains theoretically important; the present data simply indicate that it should not be assumed to explain business self-evaluation directly.

### 3.12 Theoretical implications

The most important theoretical implication is that perceived entrepreneurial role isolation can serve as a bridge between entrepreneurship research and occupational health psychology. Entrepreneurship research has increasingly recognized the psychological complexity of entrepreneurial work, including wellbeing, stress, autonomy, and identity (Stephan, 2018; Wiklund et al., 2019). Occupational health psychology has emphasized strain, recovery, work addiction, and detachment (Clark et al., 2016; Sonnentag and Fritz, 2015). Role isolation connects these domains: it is strongly associated with work addiction risk, and it is the most consistent correlate of lower perceived business success in the present data. Section 2.3 outlined informational, affective, and instrumental mechanisms through which role isolation could relate to business self-evaluation. The present design cannot adjudicate among them. What the robustness pattern does establish is that the association was not accounted for by the distress-related and firm-level covariates available in this study, and this makes mechanism-differentiating research a warranted next step rather than a speculative one.

The results also refine the theoretical status of recovery in success research. Recovery theory predicts that detachment-related recovery reduces strain and supports wellbeing (Sonnentag and Fritz, 2015; Sonnentag et al., 2018), and entrepreneurship-specific diary evidence shows that stressors impair detachment through work-related rumination (Wach et al., 2021). The present findings are consistent with recovery’s relevance to maladaptive work investment but do not support a direct association with perceived business success. This distinction matters. Recovery may be a health-protective process without being a predictor of business self-evaluation, and models that conflate the two risk mis-specifying where recovery does its work. The passion findings sharpen the motivational interpretation as well. Passion quality was more informative for understanding work addiction risk than for directly explaining perceived business success. This pattern is consistent with the dualistic model (Vallerand et al., 2003) and with calls for greater differentiation in entrepreneurial passion research (Newman et al., 2020).

### 3.13 Managerial implications

The findings support four implications, each stated at the strength the evidence permits. More work should not be equated with better perceived business functioning. Work addiction risk was associated with lower perceived business success only at the bivariate and group-comparison levels and lost significance under full adjustment. Compulsive work investment is therefore better read as a marker of a broader adverse profile involving isolation, impaired recovery, and obsessive passion than as a path to stronger business self-evaluation.

Role isolation is the most defensible support target the data identify. The high-risk work addiction group differed from controls most sharply on role isolation (*d* = 0.92), and isolation was the one variable consistently associated with lower perceived business success across all specifications. If this association is not artefactual, and the robustness pattern suggests it is not, then structures that reduce role isolation are plausible candidates for supporting leaders’ business self-evaluation. Peer networks, mentoring relationships, advisory boards, and founder groups all fit this description. These structures were not tested in the present study; they are proposed as targets for intervention-oriented research rather than as evidence-based recommendations. The cross-sectional design does not license the claim that such interventions would improve perceived success. It licenses the claim that isolation is where intervention-oriented research should look first, consistent with evidence that entrepreneurs’ functioning is embedded in social and contextual conditions (Stephan, 2018; Wiklund et al., 2019).

Recovery-supportive practices remain relevant even though recovery-related leisure time did not predict perceived business success. Recovery was clearly linked to work addiction risk, and recovery-focused entrepreneurship research ties daily recovery to entrepreneurs’ mental health (Le Moal et al., 2026; Wach et al., 2021). Recovery should therefore be framed as part of sustainable entrepreneurial functioning rather than as a productivity instrument.

Leadership development should distinguish high commitment from compulsive overinvestment. Obsessive passion was closely tied to work addiction risk (OR = 1.19 per scale point) and harmonious passion to lower risk (OR = 0.90). The aim is not to reduce entrepreneurial drive but to support forms of motivation that remain integrated with recovery and social embeddedness.

### 3.14 Limitations

Several limitations bound the claims. The design is cross-sectional, which precludes causal inference and temporal ordering. Associations among work addiction risk, role isolation, recovery-related leisure time, passion, and perceived business success should not be read as directional effects. The exploratory indirect-association analyses are statistical decompositions rather than evidence of mediation over time (Maxwell and Cole, 2007; O’Laughlin et al., 2018; Spector, 2019).

All constructs were self-reported, which raises the possibility of common method bias and negative self-evaluation effects. Robustness models with distress-related controls reduce, but cannot eliminate, the concern that general affective state shaped responses (Podsakoff and Organ, 1986; Podsakoff et al., 2024). Two focal measures, role isolation and recovery-related leisure time, were single-item proxies. Role isolation was not measured with a validated entrepreneurial loneliness or social isolation scale, and recovery-related leisure time is not a validated psychological detachment measure. These measures were conceptually useful but narrower than the constructs they approximate, and the unequal reliability of single-item and multi-item predictors complicates comparisons of coefficient magnitudes across them.

The perceived business success index captures perceived economic and business success. It represents neither full multidimensional subjective entrepreneurial success nor objective or archival performance, and subjective and objective success may follow different patterns (Dijkhuizen et al., 2018). Although the focal associations were robust to the inclusion of firm size, ownership share, life-cycle stage, leadership position, and industry, these covariates were self-reported and relatively coarse. Residual confounding by unmeasured firm-level conditions therefore cannot be ruled out. A small number of implausible time-use reports were identified through the structural screening criterion. Sensitivity analyses excluding them left the conclusions unchanged, but the survey did not include formal attention-check items. The supplementary logistic model operated near conventional events-per-variable thresholds (Vittinghoff and McCulloch, 2007) and identified high-risk respondents with modest sensitivity, so its classification results should be read as descriptive of the profile rather than as a screening instrument. The sample itself bounds generalization. It is a self-selected convenience sample of Hungarian owner-managers and business leaders recruited through entrepreneur- and employer-focused channels. Entrepreneurial role expectations, support structures, and success criteria vary across institutional and cultural contexts, and extension beyond this setting requires replication.

### 3.15 Future research

Future research should use longitudinal designs to examine whether work addiction risk precedes role isolation, whether isolation shapes later business self-evaluation, or whether lower perceived success contributes to subsequent overinvestment and isolation. Objective or archival performance indicators should be incorporated to distinguish perceived business success from actual business outcomes. Validated measures of entrepreneurial loneliness, workplace loneliness, or social embeddedness would strengthen the isolation construct and would permit the informational, affective, and instrumental mechanisms sketched here to be tested against one another. Validated detachment and rumination scales would allow more precise testing of recovery-related processes, including the possibility that recovery quality rather than recovery time is what matters. Multi-source designs that combine self-reports with employee ratings, peer assessments, mentor reports, or financial records would address common method concerns directly. Cross-cultural replication is needed, and future studies should distinguish founders, owner-managers, family business leaders, and employed managers, whose experiences of work addiction, isolation, recovery, passion, and perceived success may differ. Such research would advance the agenda proposed by Wiklund et al. (2019), Stephan (2018), and Le Moal et al. (2026) toward a more contextualized understanding of entrepreneurial wellbeing, recovery, and business self-evaluation.

## 4 Conclusion

This study examined how work addiction risk, perceived entrepreneurial role isolation, recovery-related leisure time, and work passion relate to perceived business success among Hungarian owner-managers and business leaders. The central empirical finding is the consistency of the negative association between perceived role isolation and perceived business success. This association held across bivariate, multivariate, and robustness specifications, whereas work addiction risk showed no stable direct association once role conditions and motivational quality were considered. The theoretical reading of this pattern, including the informational, affective, and instrumental mechanisms, and the practical directions built on it, such as peer networks and mentoring structures, remain tentative. They were not tested directly and require longitudinal, multi-source, and intervention-oriented designs. Within these bounds, the study positions the social experience of the leadership role as a central concern for research on how entrepreneurs evaluate the condition of their businesses.

## Data Availability

The original contributions presented in the study are included in the article/Supplementary material, further inquiries can be directed to the corresponding author.
